# Supplementary material for: Neurocognitive patterns dissociating semantic processing from executive control are linked to more detailed off-task mental time travel
Source: Sci Rep. 2020 Jul 17;10:11904. doi: 10.1038/s41598-020-67605-2 (PMC7368037; doi:10.1038/s41598-020-67605-2)
Supplement: Supplementary file 1 — Supplementary information 1 [file 41598_2020_67605_MOESM1_ESM.docx]

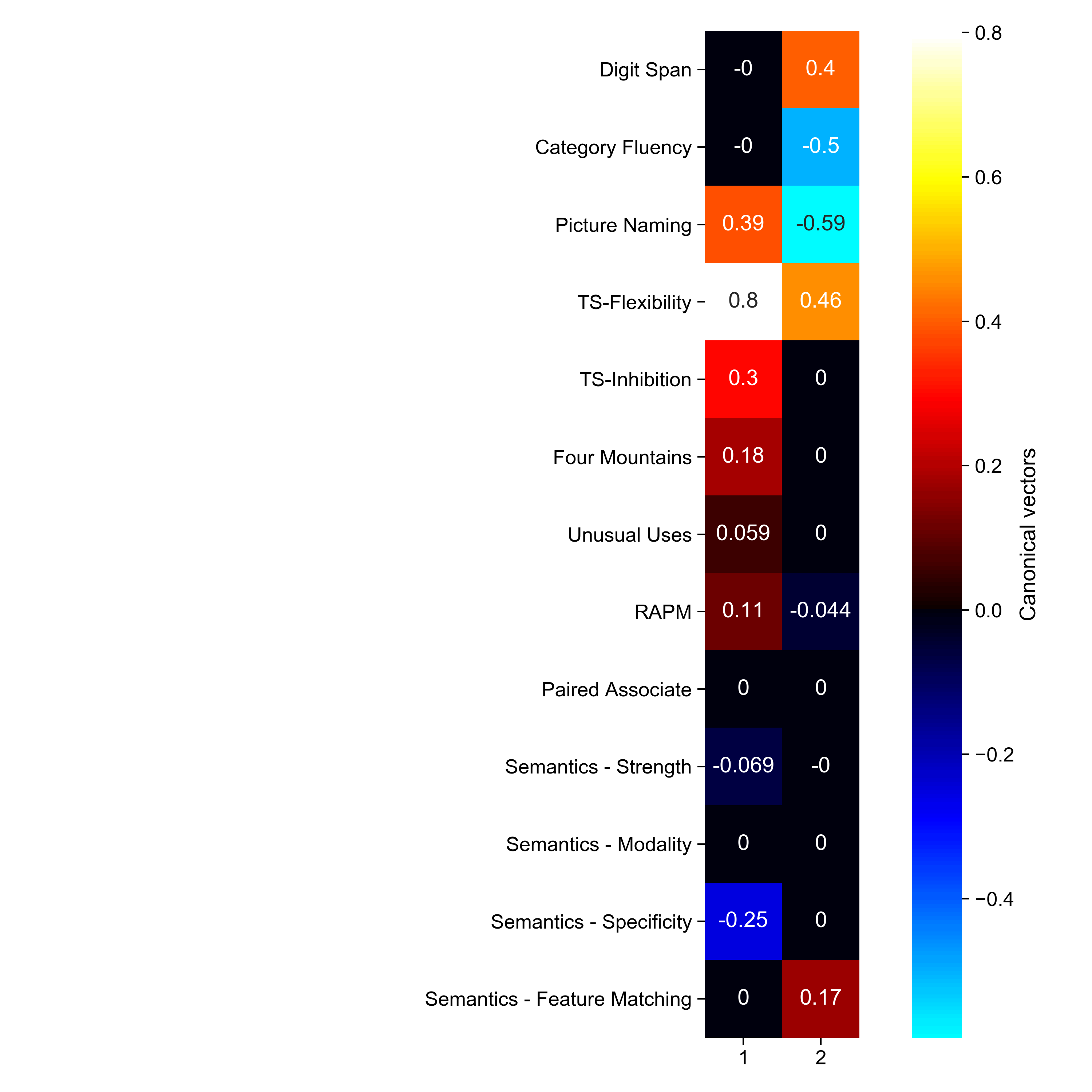
Heat maps of the cognitive task component in the two significant modes

Full statistic table of the multiple multivariate regression

Multivariate linear model

==============================================================

--------------------------------------------------------------

Mode 1 Value Num DF Den DF F Value Pr > F

--------------------------------------------------------------

Wilks' lambda 0.9518 13.0000 164.0000 0.6390 0.8186

Pillai's trace 0.0482 13.0000 164.0000 0.6390 0.8186

Hotelling-Lawley trace 0.0506 13.0000 164.0000 0.6390 0.8186

Roy's greatest root 0.0506 13.0000 164.0000 0.6390 0.8186

--------------------------------------------------------------

--------------------------------------------------------------

Mode 2 Value Num DF Den DF F Value Pr > F

-------------------------------------------------------------

Wilks' lambda 0.8147 13.0000 164.0000 2.8694 0.0009

Pillai's trace 0.1853 13.0000 164.0000 2.8694 0.0009

Hotelling-Lawley trace 0.2275 13.0000 164.0000 2.8694 0.0009

Roy's greatest root 0.2275 13.0000 164.0000 2.8694 0.0009

==============================================================

OLS Regression Results

==============================================================================

Dep. Variable: MWQ_Deliberate R-squared: 0.007

Model: OLS Adj. R-squared: -0.004

Method: Least Squares F-statistic: 0.6159

Date: Tue, 10 Sep 2019 Prob (F-statistic): 0.541

Time: 15:48:38 Log-Likelihood: -250.70

No. Observations: 178 AIC: 507.4

Df Residuals: 175 BIC: 516.9

Df Model: 2

Covariance Type: nonrobust

===============================================================================

coef std err t P>|t| [0.025 0.975]

-------------------------------------------------------------------------------

Intercept 0.0047 0.075 0.063 0.950 -0.143 0.152

Mode 1 -0.0138 0.042 -0.327 0.744 -0.097 0.070

Mode 2 -0.0440 0.042 -1.047 0.297 -0.127 0.039

==============================================================================

Omnibus: 3.950 Durbin-Watson: 2.120

Prob(Omnibus): 0.139 Jarque-Bera (JB): 4.473

Skew: 0.128 Prob(JB): 0.107

Kurtosis: 3.733 Cond. No. 1.81

==============================================================================

OLS Regression Results

==============================================================================

Dep. Variable: MWQ_Detailed R-squared: 0.043

Model: OLS Adj. R-squared: 0.032

Method: Least Squares F-statistic: 3.968

Date: Tue, 10 Sep 2019 Prob (F-statistic): 0.0206

Time: 15:48:38 Log-Likelihood: -254.25

No. Observations: 178 AIC: 514.5

Df Residuals: 175 BIC: 524.1

Df Model: 2

Covariance Type: nonrobust

===============================================================================

coef std err t P>|t| [0.025 0.975]

-------------------------------------------------------------------------------

Intercept 0.0098 0.076 0.129 0.898 -0.141 0.160

Mode 1 -0.0608 0.043 -1.409 0.161 -0.146 0.024

Mode 2 -0.1021 0.043 -2.381 0.018 -0.187 -0.017

==============================================================================

Omnibus: 1.395 Durbin-Watson: 2.029

Prob(Omnibus): 0.498 Jarque-Bera (JB): 1.244

Skew: -0.033 Prob(JB): 0.537

Kurtosis: 2.596 Cond. No. 1.81

==============================================================================

OLS Regression Results

==============================================================================

Dep. Variable: MWQ_Emotion R-squared: 0.006

Model: OLS Adj. R-squared: -0.005

Method: Least Squares F-statistic: 0.5309

Date: Tue, 10 Sep 2019 Prob (F-statistic): 0.589

Time: 15:48:38 Log-Likelihood: -245.02

No. Observations: 178 AIC: 496.0

Df Residuals: 175 BIC: 505.6

Df Model: 2

Covariance Type: nonrobust

===============================================================================

coef std err t P>|t| [0.025 0.975]

-------------------------------------------------------------------------------

Intercept -0.0903 0.072 -1.246 0.214 -0.233 0.053

Mode 1 -0.0188 0.041 -0.459 0.647 -0.100 0.062

Mode 2 0.0383 0.041 0.940 0.348 -0.042 0.119

==============================================================================

Omnibus: 3.980 Durbin-Watson: 1.421

Prob(Omnibus): 0.137 Jarque-Bera (JB): 3.605

Skew: 0.338 Prob(JB): 0.165

Kurtosis: 3.168 Cond. No. 1.81

==============================================================================

OLS Regression Results

==============================================================================

Dep. Variable: MWQ_Evolving R-squared: 0.006

Model: OLS Adj. R-squared: -0.005

Method: Least Squares F-statistic: 0.5182

Date: Tue, 10 Sep 2019 Prob (F-statistic): 0.596

Time: 15:48:38 Log-Likelihood: -254.02

No. Observations: 178 AIC: 514.0

Df Residuals: 175 BIC: 523.6

Df Model: 2

Covariance Type: nonrobust

===============================================================================

coef std err t P>|t| [0.025 0.975]

-------------------------------------------------------------------------------

Intercept -0.0019 0.076 -0.025 0.980 -0.152 0.149

Mode 1 -0.0400 0.043 -0.928 0.355 -0.125 0.045

Mode 2 0.0195 0.043 0.455 0.650 -0.065 0.104

==============================================================================

Omnibus: 2.454 Durbin-Watson: 1.890

Prob(Omnibus): 0.293 Jarque-Bera (JB): 2.111

Skew: -0.159 Prob(JB): 0.348

Kurtosis: 3.429 Cond. No. 1.81

==============================================================================

OLS Regression Results

==============================================================================

Dep. Variable: MWQ_Task R-squared: 0.019

Model: OLS Adj. R-squared: 0.008

Method: Least Squares F-statistic: 1.719

Date: Tue, 10 Sep 2019 Prob (F-statistic): 0.182

Time: 15:48:38 Log-Likelihood: -253.59

No. Observations: 178 AIC: 513.2

Df Residuals: 175 BIC: 522.7

Df Model: 2

Covariance Type: nonrobust

===============================================================================

coef std err t P>|t| [0.025 0.975]

-------------------------------------------------------------------------------

Intercept 0.0199 0.076 0.261 0.794 -0.130 0.170

Mode 1 -0.0288 0.043 -0.670 0.504 -0.114 0.056

Mode 2 0.0749 0.043 1.754 0.081 -0.009 0.159

==============================================================================

Omnibus: 0.220 Durbin-Watson: 2.069

Prob(Omnibus): 0.896 Jarque-Bera (JB): 0.227

Skew: -0.082 Prob(JB): 0.893

Kurtosis: 2.939 Cond. No. 1.81

==============================================================================

OLS Regression Results

==============================================================================

Dep. Variable: MWQ_Future R-squared: 0.020

Model: OLS Adj. R-squared: 0.009

Method: Least Squares F-statistic: 1.797

Date: Tue, 10 Sep 2019 Prob (F-statistic): 0.169

Time: 15:48:39 Log-Likelihood: -255.02

No. Observations: 178 AIC: 516.0

Df Residuals: 175 BIC: 525.6

Df Model: 2

Covariance Type: nonrobust

===============================================================================

coef std err t P>|t| [0.025 0.975]

-------------------------------------------------------------------------------

Intercept -0.0268 0.077 -0.350 0.727 -0.178 0.124

Mode 1 -0.0267 0.043 -0.615 0.539 -0.112 0.059

Mode 2 -0.0761 0.043 -1.768 0.079 -0.161 0.009

==============================================================================

Omnibus: 2.882 Durbin-Watson: 2.010

Prob(Omnibus): 0.237 Jarque-Bera (JB): 2.456

Skew: -0.264 Prob(JB): 0.293

Kurtosis: 3.230 Cond. No. 1.81

==============================================================================

OLS Regression Results

==============================================================================

Dep. Variable: MWQ_Habit R-squared: 0.031

Model: OLS Adj. R-squared: 0.020

Method: Least Squares F-statistic: 2.834

Date: Tue, 10 Sep 2019 Prob (F-statistic): 0.0615

Time: 15:48:39 Log-Likelihood: -250.56

No. Observations: 178 AIC: 507.1

Df Residuals: 175 BIC: 516.7

Df Model: 2

Covariance Type: nonrobust

===============================================================================

coef std err t P>|t| [0.025 0.975]

-------------------------------------------------------------------------------

Intercept -0.0090 0.075 -0.121 0.904 -0.157 0.138

Mode 1 -0.0216 0.042 -0.512 0.609 -0.105 0.062

Mode 2 -0.0967 0.042 -2.303 0.022 -0.180 -0.014

==============================================================================

Omnibus: 4.204 Durbin-Watson: 2.035

Prob(Omnibus): 0.122 Jarque-Bera (JB): 4.907

Skew: 0.131 Prob(JB): 0.0860

Kurtosis: 3.770 Cond. No. 1.81

==============================================================================

OLS Regression Results

==============================================================================

Dep. Variable: MWQ_Images R-squared: 0.003

Model: OLS Adj. R-squared: -0.009

Method: Least Squares F-statistic: 0.2530

Date: Tue, 10 Sep 2019 Prob (F-statistic): 0.777

Time: 15:48:39 Log-Likelihood: -257.59

No. Observations: 178 AIC: 521.2

Df Residuals: 175 BIC: 530.7

Df Model: 2

Covariance Type: nonrobust

===============================================================================

coef std err t P>|t| [0.025 0.975]

-------------------------------------------------------------------------------

Intercept -0.0351 0.078 -0.451 0.653 -0.189 0.118

Mode 1 -0.0109 0.044 -0.247 0.805 -0.098 0.076

Mode 2 -0.0287 0.044 -0.657 0.512 -0.115 0.058

==============================================================================

Omnibus: 3.184 Durbin-Watson: 2.163

Prob(Omnibus): 0.204 Jarque-Bera (JB): 3.242

Skew: -0.308 Prob(JB): 0.198

Kurtosis: 2.757 Cond. No. 1.81

==============================================================================

OLS Regression Results

==============================================================================

Dep. Variable: MWQ_People R-squared: 0.005

Model: OLS Adj. R-squared: -0.006

Method: Least Squares F-statistic: 0.4832

Date: Tue, 10 Sep 2019 Prob (F-statistic): 0.618

Time: 15:48:39 Log-Likelihood: -253.83

No. Observations: 178 AIC: 513.7

Df Residuals: 175 BIC: 523.2

Df Model: 2

Covariance Type: nonrobust

===============================================================================

coef std err t P>|t| [0.025 0.975]

-------------------------------------------------------------------------------

Intercept -0.0364 0.076 -0.479 0.633 -0.187 0.114

Mode 1 -0.0288 0.043 -0.669 0.505 -0.114 0.056

Mode 2 -0.0297 0.043 -0.694 0.489 -0.114 0.055

==============================================================================

Omnibus: 1.668 Durbin-Watson: 2.039

Prob(Omnibus): 0.434 Jarque-Bera (JB): 1.682

Skew: -0.230 Prob(JB): 0.431

Kurtosis: 2.877 Cond. No. 1.81

==============================================================================

OLS Regression Results

==============================================================================

Dep. Variable: MWQ_Past R-squared: 0.018

Model: OLS Adj. R-squared: 0.007

Method: Least Squares F-statistic: 1.613

Date: Tue, 10 Sep 2019 Prob (F-statistic): 0.202

Time: 15:48:39 Log-Likelihood: -257.21

No. Observations: 178 AIC: 520.4

Df Residuals: 175 BIC: 530.0

Df Model: 2

Covariance Type: nonrobust

===============================================================================

coef std err t P>|t| [0.025 0.975]

-------------------------------------------------------------------------------

Intercept 0.0023 0.078 0.030 0.976 -0.151 0.155

Mode 1 -0.0097 0.044 -0.222 0.825 -0.096 0.077

Mode 2 -0.0773 0.044 -1.772 0.078 -0.163 0.009

==============================================================================

Omnibus: 3.157 Durbin-Watson: 2.119

Prob(Omnibus): 0.206 Jarque-Bera (JB): 3.176

Skew: -0.319 Prob(JB): 0.204

Kurtosis: 2.856 Cond. No. 1.81

==============================================================================

OLS Regression Results

==============================================================================

Dep. Variable: MWQ_Self R-squared: 0.020

Model: OLS Adj. R-squared: 0.008

Method: Least Squares F-statistic: 1.746

Date: Tue, 10 Sep 2019 Prob (F-statistic): 0.178

Time: 15:48:39 Log-Likelihood: -255.28

No. Observations: 178 AIC: 516.6

Df Residuals: 175 BIC: 526.1

Df Model: 2

Covariance Type: nonrobust

===============================================================================

coef std err t P>|t| [0.025 0.975]

-------------------------------------------------------------------------------

Intercept -0.0142 0.077 -0.185 0.854 -0.166 0.137

Mode 1 0.0213 0.043 0.491 0.624 -0.064 0.107

Mode 2 -0.0785 0.043 -1.821 0.070 -0.164 0.007

==============================================================================

Omnibus: 10.753 Durbin-Watson: 1.891

Prob(Omnibus): 0.005 Jarque-Bera (JB): 10.918

Skew: -0.566 Prob(JB): 0.00426

Kurtosis: 3.436 Cond. No. 1.81

==============================================================================

OLS Regression Results

==============================================================================

Dep. Variable: MWQ_Vivid R-squared: 0.003

Model: OLS Adj. R-squared: -0.009

Method: Least Squares F-statistic: 0.2284

Date: Tue, 10 Sep 2019 Prob (F-statistic): 0.796

Time: 15:48:39 Log-Likelihood: -255.99

No. Observations: 178 AIC: 518.0

Df Residuals: 175 BIC: 527.5

Df Model: 2

Covariance Type: nonrobust

===============================================================================

coef std err t P>|t| [0.025 0.975]

-------------------------------------------------------------------------------

Intercept -0.0136 0.077 -0.176 0.860 -0.166 0.139

Mode 1 0.0015 0.044 0.035 0.972 -0.084 0.088

Mode 2 -0.0293 0.043 -0.676 0.500 -0.115 0.056

==============================================================================

Omnibus: 0.556 Durbin-Watson: 1.900

Prob(Omnibus): 0.757 Jarque-Bera (JB): 0.690

Skew: 0.064 Prob(JB): 0.708

Kurtosis: 2.723 Cond. No. 1.81

==============================================================================

OLS Regression Results

==============================================================================

Dep. Variable: MWQ_Words R-squared: 0.010

Model: OLS Adj. R-squared: -0.001

Method: Least Squares F-statistic: 0.8985

Date: Tue, 10 Sep 2019 Prob (F-statistic): 0.409

Time: 15:48:39 Log-Likelihood: -255.23

No. Observations: 178 AIC: 516.5

Df Residuals: 175 BIC: 526.0

Df Model: 2

Covariance Type: nonrobust

===============================================================================

coef std err t P>|t| [0.025 0.975]

-------------------------------------------------------------------------------

Intercept 0.0423 0.077 0.551 0.582 -0.109 0.194

Mode 1 -0.0577 0.043 -1.329 0.186 -0.143 0.028

Mode 2 0.0099 0.043 0.229 0.819 -0.075 0.095

==============================================================================

Omnibus: 3.456 Durbin-Watson: 2.191

Prob(Omnibus): 0.178 Jarque-Bera (JB): 3.334

Skew: -0.335 Prob(JB): 0.189

Kurtosis: 2.974 Cond. No. 1.81

==============================================================================

List of terms in the neurosynth analysis

Neurosynth clusters the keywords into 50 topics: <https://raw.githubusercontent.com/neurosynth/neurosynth-web/master/data/topics/keys/v4-topics-50.txt>

The clustered results has no labels. After eliminating unrelated topics, the following topics were kept for the analysis:

Index Assigned label

0 motion perception

1 object recognition

2 cued attention

3 multisensory processing

5 facial recognition

8 inhibition error

11 motion and visuospatial memory

12 episodic memory

15 eye movement

17 social cognition

20 reading

22 working memory

23 face/affective processing

25 feedback based learning

29 reward based decision making

32 auditory processing

36 sentences comprehension

40 emotion regulation

41 visual attention

42 numerical cognition

44 verbal semantics

47 resolve conflicts

48 pain

49 sesnorimotor
